# Measuring pulmonary oxygen uptake kinetics: Contemporary perspectives

**DOI:** 10.1113/EP091657

**Published:** 2023-12-29

**Authors:** Harry B. Rossiter, David C. Poole

**Affiliations:** ^1^ Institute of Respiratory Medicine and Exercise Physiology Division of Respiratory and Critical Care Physiology and Medicine The Lundquist Institute for Biomedical Innovation at Harbor‐UCLA Medical Center Torrance California USA; ^2^ Department of Kinesiology Kansas State University Manhattan Kansas USA; ^3^ Department of Anatomy & Physiology Kansas State University Manhattan Kansas USA

**Keywords:** breath‐to‐breath variability, energetics, exercise, exercise testing, lung gas stores, oxygen uptake kinetics, parameter extraction, pulmonary gas exchange

1

As championed by Brian J. Whipp, resolution of pulmonary oxygen uptake (${\dot{V}}_{O_{2}}$) kinetics following exercise onset provides a unique and non‐invasive window into muscle energetics, mitochondrial function and the mechanistic bases for exercise tolerance (reviewed by Poole & Jones, 2012; Rossiter, 2011). In theory and practice, beyond that time taken for blood transit from the exercising muscles to reach the lungs (i.e., phase I), the close‐to‐exponential so‐called ‘primary’ or ‘fundamental’ ${\dot{V}}_{O_{2}}$ response (i.e., phase II) at the blood–gas barrier in the lung closely reflects that across the exercising muscles (reviewed by Poole & Jones, 2012; Rossiter, 2011).

Notwithstanding the above, because breathing is an inherently ‘noisy’ process, with high breath‐to‐breath variability inducing wide swings in intrathoracic pressure, coupled with a pulsatile pulmonary blood flow and complex dynamics of lung volumes and gas stores (primarily O_2_ and CO_2_), modelling of the underlying gas exchange responses and accurate parameter extraction are challenging. In this issue of *Experimental Physiology*, Francescato and Cettolo (2023) recognize the importance of accounting for altered lung gas stores upon changes in exercise power output. To do this, they use inspiratory and expiratory flow and concentration profiles analysed using their ‘independent‐breath’ algorithm. Their results demonstrate that, by accounting for breath‐to‐breath differences in lung gas stores, the robustness of the gas exchange measurement is increased using the independent‐breath algorithm compared with an ‘expiration only’ algorithm; the latter is based on the pioneering work of Grønlund and (separately) Auchincloss, and can be applied only post hoc, once all the data have been collected (reviewed by Girardi et al., 2023). The increased accuracy of alveolar gas exchange measurements by the independent‐breath algorithm was achieved principally by means of a shorter modelled time delay of the rise in ${\dot{V}}_{O_{2}}$ and pulmonary carbon dioxide output (${\dot{V}}_{{\mathrm{CO}}_{2}}$). Hence, the ${\dot{V}}_{O_{2}}$ mean response time (MRT) is consistently shorter using the independent‐breath algorithm by ∼10%, and the estimated O_2_ deficit (O_2_ deficit = ∆${\dot{V}}_{O_{2}}$ × MRT) is likewise smaller.

Francescato and Cettolo's (2023) findings largely support assertions from their own previous work but strengthen their conclusions by using 10 bouts of exercise to reduce sample‐to‐sample variability and enhance the confidence in the fitted parameter values. Readers interested in the insight provided by pulmonary ${\dot{V}}_{O_{2}}$ kinetics for muscle ${\dot{V}}_{O_{2}}$ need not shred their ${\dot{V}}_{O_{2}}$ kinetics pdf libraries, however, because the primary (phase II) time constant measured by either algorithm did not differ when the fitting window was constrained to start between 16 and 29 s (Fig. 2 in the paper by Francescato & Cettolo, 2023); it is, and was, common practice in the field to remove the influence of phase I by removing the first ∼20 s of ${\dot{V}}_{O_{2}}$ (Benson et al., 2017; Whipp et al., 1982). As such, foundational prior work remains a robust scaffold upon which to build future discovery. Nevertheless, the accuracy and precision of ${\dot{V}}_{O_{2}}$ kinetics analysis by the independent‐breath algorithm was not pitted against the array of other approaches that are currently in use in academic laboratories or available in clinical instruments (Girardi et al., 2023); therefore, we remain uncertain about the magnitude of potential differences among these various approaches.

A ∼10% reduction in the estimated O_2_ deficit represents a welcome improvement in the accuracy of measurement (see arguments by Poole & Jones (2017) advocating for the most precise and accurate gas exchange methods available). This improved accuracy results essentially from tracking the increase in lung O_2_ storage caused, most probably, predominantly by a drop in end‐expiratory lung volume occurring within the first few breaths after an abrupt increase in exercise power output. However, this approach presents a fundamental challenge to our understanding of what constitutes ‘a breath’. A breath, or a single breathing cycle, as traditionally defined, is the time interval between the start of two consecutive inspirations. The independent‐breath algorithm redefines ‘a breath’, for ${\dot{V}}_{O_{2}}$, as the time between two equal ratios of fractional concentration of O_2_ and N_2_ (i.e., the FO2/FO2FN2FN2 ratio). In similar fashion, the time between two equal ratios of FCO2/FCO2FN2FN2 defines a breath for ${\dot{V}}_{{\mathrm{CO}}_{2}}$. And therein lies the challenge. Ventilation, tidal volume or breathing frequency can now differ on a ‘breath‐by‐breath’ basis, depending on the respiratory gas considered. More perplexing still, it is possible that the FO2/FO2FN2FN2 ratio (or FCO2/FCO2FN2FN2 ratio) might not be equal in two consecutive inspirations, and therefore two breaths become one under the more precise and accurate independent‐breath approach to gas exchange analysis.

Increasing the signal‐to‐noise ratio to improve characterization of clinically important exercise testing variables is a fundamental benefit to gas exchange algorithms that accounts more completely for changes in lung gas stores (Girardi et al., 2023). However, without a gold standard for alveolar gas exchange, it is hard to assess rigorously the relative strengths and weaknesses of the various approaches available. Furthermore, the requirement to redefine what is meant by ‘a breath’, through adoption of the independent‐breath algorithm, could fundamentally change the clinical interpretation of exercise testing breathing responses in ways that are currently unknown. In either case, it is incumbent on the research community to bridge these significant knowledge gaps, while simultaneously striving to develop the most precise and accurate measurement methods, in the manner that Francescato and Cettolo (2023) have shown.

## AUTHOR CONTRIBUTIONS

Both authors approve of the final version of the manuscript and agree to be accountable for all aspects of thework, ensuring that questions related to the accuracy or integrity are appropriately investigated and resolved. All persons designated as authors qualify for authorship, and all those that quality are listed.

## CONFLICT OF INTEREST

None declared.

## FUNDING INFORMATION

No funding was received for this work.
